# Spent Coffee Grounds as an Adsorbent for Malathion and Chlorpyrifos—Kinetics, Thermodynamics, and Eco-Neurotoxicity

**DOI:** 10.3390/foods12122397

**Published:** 2023-06-16

**Authors:** Vedran Milanković, Tamara Tasić, Milica Pejčić, Igor Pašti, Tamara Lazarević-Pašti

**Affiliations:** 1 VINČA Institute of Nuclear Sciences—National Institute of the Republic of Serbia, University of Belgrade, Mike Petrovica Alasa 12–14, 11000 Belgrade, Serbia; vedran.milankovic@vin.bg.ac.rs (V.M.); tamara.tasic@vin.bg.ac.rs (T.T.); milica.pejcic@vin.bg.ac.rs (M.P.); 2 Faculty of Physical Chemistry, University of Belgrade, Studentski Trg 12–16, 11158 Belgrade, Serbia; igor@ffh.bg.ac.rs

**Keywords:** biowaste, spent coffee grounds, organophosphates, pesticides, food processing, adsorption

## Abstract

Coffee is one of the most popular beverages, with around 10.5 million tons manufactured annually. The same amount of spent coffee grounds (SCGs) might harm the environment if disposed of carelessly. On the other hand, pesticide contamination in food and biowaste is a rising problem. Because pesticides are hazardous and can cause serious health consequences, it is critical to understand how they interact with food biowaste materials. However, it is also a question if biowaste can be used to remediate rising pesticide residues in the environment. This study investigated the interactions of SCGs with the organophosphate pesticides malathion (MLT) and chlorpyrifos (CHP) and addressed the possibility of using SCGs as adsorbents for the removal of these pesticides from water and fruit extracts. The kinetics of MLT and CHP adsorption on SCGs fits well with the pseudo-first-order kinetic model. The Langmuir isotherm model best describes the adsorption process, giving the maximal adsorption capacity for MLT as 7.16 mg g^−1^ and 7.00 mg g^−1^ for CHP. Based on the thermodynamic analysis, it can be deduced that MLT adsorption on SCGs is exothermic, while CHP adsorption is an endothermic process. The adsorption efficiency of MLT and CHP using SCGs in a complicated matrix of fruit extracts remained constant. The neurotoxicity results showed that no more toxic products were formed during adsorption, indicating that SCGs are a safe-to-use adsorbent for pesticide removal in water and fruit extracts.

## 1. Introduction

Food biowaste represents an organic waste material derived from food processing, preparation, and serving activities, including agricultural by-products and kitchen scraps [1]. It is becoming a significant global issue, as the components of biowaste release CO_2_ and other greenhouse gases in the process of breaking down, in addition to the economic and moral factors of throwing food away. Up to 30% of food biowaste globally is leftovers from restaurants, grocery stores, and households [2]. 

Coffee is one of the most popular beverages, with around 10.5 million tons produced yearly [3]. However, the same amount of spent coffee grounds (SCGs) could negatively affect the environment if disposed of improperly. When exposed to different environmental influences, coffee grounds release nitrogen and carbon dioxide into the atmosphere [4,5]. Nitrogen reacts with oxygen to form smog and ozone, which are both detrimental to the environment, while carbon dioxide is a greenhouse gas [6]. If disposed of in water, spent coffee grounds could increase acidity, thus decreasing oxygenation. These factors could lead to an increase in algae and bacteria growth [7]. To reduce the negative environmental impact of spent coffee grounds, they should be composted, reused, or recycled instead of thrown away. 

SCGs have recently been gaining attention for their potential uses in many fields, including gardening, energy production, and environmental protection. Once recycled, they can play essential roles in many industries, especially energy production, agroecology, and sustainable practices [8,9]. Spent coffee grounds are composed mainly of cellulose, lignin, and carbohydrates [10,11]. These properties make it a suitable adsorbent material that can be used to remove heavy metals, dyes, pesticides, and other pollutants from water, while Pujol et al. [10] particularly emphasized SCGs as a potential sorbent for hydrophobic pollutants.

Pesticides are one of the most widely used substances around the world. Although they are essential in controlling pests and protecting crops, they can also cause significant harm to human health and the environment if used excessively or carelessly [12]. Organophosphate pesticides (OPs) are widely used and efficient but very harmful for different species. OPs’ toxicity is connected to their ability to inhibit acetylcholinesterase (AChE) activity. AChE is an enzyme with a key role in neurotransmission in animals. Its inhibition severely affects humans and other non-targeted species, such as bees [13,14]. Therefore, developing effective methods to remove OPs from the environment is essential. Except for the central (thio)phosphate moiety common for all OPs, they can have very different structures. For this work, we considered two OPs, chlorpyrifos and malathion. CHP has an aromatic moiety, while MLT is an aliphatic molecule. Thus, the parallel investigation of these two pesticides allows us to better understanding of the role of the adsorbent structure and chemistry in the adsorption of structurally different OPs.

Chlorpyrifos (CHP) is an organophosphate pesticide commonly used to control pests on fruits and vegetables, such as apples, oranges, lemons, limes, peaches, nectarines, bananas, grapes, tomatoes, peppers, and strawberries [15]. It is typically applied through aerial spraying or ground application and is highly regulated due to its toxicity [16]. Using chlorpyrifos in mint cultivation can help protect the mint crop against aphids, whiteflies, and spider mites [17]. China produces more than 30% of the world’s supply of chlorpyrifos, followed by the United States and India. In Europe, the highest production of chlorpyrifos is located in France and the Netherlands. It is highly regulated due to its potential toxicity. Improper use of chlorpyrifos can lead to health problems in humans and other animals, so careful selection and application of this pesticide are necessary.

Malathion (MLT) is an insecticide that is widely used in lemon cultivation [18,19]. Like other organophosphate pesticides, malathion exerts its effects by affecting the nervous system of the pests [20]. Although it is moderately toxic to humans, it is essential to use malathion responsibly, as overuse of this pesticide can harm other animals’ health and the environment.

The presence of pesticides in food and biowaste materials is a growing concern. Due to their toxic nature and potential health impacts, it is crucial to understand how pesticides interact with food biowaste materials. Adsorption is one of the most widely studied processes for remediation by which a pollutant, such as a pesticide, binds to a surface and is subsequently removed. This surface binding can be either physical or chemical [21]. Physical adsorption occurs when adsorbate molecules are attracted to the surface of the adsorbent material without forming chemical bonds [22]. Chemical adsorption, on the other hand, occurs when adsorbate molecules chemically react with the surface of the adsorbent material [21]. One of the most significant benefits is that adsorption reduces pollutants entering soil, water, and air systems. Moreover, it is cost-effective and does not require complex technologies or processes [23].

Several factors influence the adsorption of pesticides on food biowaste materials. Some of them are the surface properties of biowaste, such as surface functional groups, the type of biowaste material, the pH of the solution, and the chemical and physical properties of the pesticide [24]. Adsorption effectiveness also depends on the pesticide concentration in the solution, the temperature, and the time in contact with the biowaste material [25,26]. The adsorption of pesticides on food biowaste materials has many important implications, leading to the possible accumulation of pesticides or indicating the potential applicability of biowaste in pesticide remediation. 

This study investigated the potential of using SCGs as an adsorbent for malathion and chlorpyrifos removal from water and fruit extracts. Our goal was to use SCGs as received, with minimal additional treatment, and without an intense use of energy and/or chemicals and release of greenhouse gases. In this way, we aimed to investigate the possible accumulation of OPs in waste SCGs and assess the direct reuse of SCGs in compliance with contemporary environmental protection strategies [27]. First, the results of the physicochemical characterization of SCGs are presented. Then, CHP and MLT adsorption on SCGs in water is analyzed. The kinetics and thermodynamics of MLT and CHP adsorption on SCGs are discussed. The adsorption of OP from realistic samples (plant extract) on SCGs is also addressed to determine the possibility of using SCGs as an adsorbent and detect if the complex matrixes influence the process. In addition, the eco-neurotoxicity of the spiked fruit extracts was monitored during the remediation process to determine if more toxic products were formed, such as pesticides’ oxo-forms. Finally, this method’s feasibility and potential benefits in food processing are discussed. At the time of submitting this article, to the best of our knowledge, no research articles were dedicated to the investigation of remediation of MLT and CHP using SCGs as a sustainable material. Moreover, in this study, the eco-neurotoxicity testing of the samples before and after the adsorption process was performed for the first time.

## 2. Materials and Methods

### 2.1. Adsorbent Preparation

Coffee (purchased from the local market, 80% Arabica and 20% Robusta) was brewed (treated with boiling water) and left for 2 h at room temperature until it became to cold. Next, the coffee grounds were separated with filtration and left to dry at room temperature for 24 h. Then, in order to release the leftover moisture, the obtained spent coffee grounds were dried in the oven at 80 °C for 1 h and ground for 15 min, using an agate stone mortar. Finally, 100 mg of the material was rinsed with 50 mL of HCl, NaOH (Centrochem, Stara Pazova, Srbija), and H_2_O and eventually dispersed in 50 mL 50% EtOH (J.T. Baker, Phillipsburg, NJ, USA), obtaining the stock dispersion concentration of 2 mg mL^−1^.

### 2.2. Adsorbent Characterization 

The fractional sieving method was used to determine the particle size distribution in the SCG sample (sieve sizes 200, 100, and 63 μm; ROTH, Karlsruhe, Germany). For the investigation of samples’ morphology and elemental composition, Scanning Electron Microscopy (SEM) and Energy Dispersive X-Ray Analysis (EDX) were performed using a scanning electron microscope PhenomProX (Thermo Fisher Scientific, Waltham, MA, USA). Fourier-transform infrared (FTIR) spectra were recorded using a Nicolet iS20 FT-IR spectrophotometer (Thermo Fisher Scientific, Waltham, MA, USA). The applied wavenumber range was from 4000 to 500 cm^−1^, with 64 scans and 4 cm^−1^. A TA Instruments SDT 2960 thermoanalytical device (TA Instruments, Inc. New Castle, DE, USA) was used for thermogravimetric analysis (TGA). The analysis was performed with a heating rate of 10 °C min^−1^ up to the temperature of 900 °C and under purging helium gas (Messer, Belgrade, Serbia). For the temperature-programmed desorption (TPD) analysis, the SCG sample was heated in a vacuum (starting pressure 1 × 10^−7^ mbar), with a constant heating rate of 10 °C min^−1^, from room temperature to 1000 °C (on the heater). Desorbed gaseous products were detected using a quadrupole mass spectrometer EXTORR XT300 (Extorr Inc., New Kensington, PA, USA).

### 2.3. Adsorption Experiments

A mixture of 1 mL SCG stock dispersion and the designated amounts of chlorpyrifos and malathion (Pestanal, Sigma Aldrich, Søborg, Denmark) stock solutions (made in 50 vol.% ethanol in water) was made to deliver the targeted concentration of adsorbent and OP. After that, the mixtures were put in a laboratory shaker and left for the specified period. Subsequently, they underwent centrifugation at 14,500 rpm, with their supernatant filtered through a nylon membrane. Ultra-Performance Liquid Chromatography (UPLC) analysis was then conducted to determine the concentrations of CHP and MLT with a Waters ACQUITY UPLC system and a Photodiode array (PDA) detector managed by Empower 3 software. An ACQUITY UPLC™ BEH C18 column (1.7 μm, 100 mm × 2.1 mm) was used under isocratic conditions of 10% acetonitrile (J.T. Baker, Phillipsburg, NJ, USA) in water (*v*/*v*) for mobile phase A and pure acetonitrile for mobile phase B. The eluent flow rate was 0.2 mL min^−1^ in all cases, with an injection volume of 5 µL. The mobile phase used for chlorpyrifos contained 20% A and 80% B, and 40% A and 60% B for malathion. The retention time for malathion was 3.2 min, and for chlorpyrifos, it was 2.7 min. Both OPs were detected at 200 nm. Control experiments were performed identically but without an adsorbent.

### 2.4. Plant Extracts Preparation

Lemon juice and mint extract were used as food samples. Lemon juice was made by squeezing one lemon (75 g) and diluting it with 500 mL of tap water (pH = 4.5) before adding MLT to the desired concentration. Next, the sample was filtered. The mint extract was prepared by mixing 7 g of *Mentha spicata* leaves with 45 mL of 50% ethanol and leaving it for 72 h at room temperature; this extract was then filtered and diluted with 200 mL of 50% ethanol (pH = 6.0). CHP was then added in the required amount, and the resulting solution was further filtered through a nylon filter. The prepared extracts were used for sample analysis.

### 2.5. Eco-Neurotoxicity Assessment

The physiological effects of the treated solutions were analyzed using AChE inhibition measurements. By employing modified Ellman’s procedure [28,29], 2.5 IU commercially purified AChE (Sigma Aldrich, Taufkirchen, Germany) from an electric eel was exposed to the OP solutions in 50 mM phosphate buffer, pH = 8.0, at 37 °C in a 0.650 mL final volume. The combination of acetylcholine-iodide (AChI, Sigma Aldrich, Taufkirchen, Germany) and DTNB (Sigma Aldrich, Taufkirchen, Germany) as a chromogenic reagent triggered the enzymatic reaction. The reaction was allowed to proceed for 8 min before being stopped with 10% sodium dodecyl sulfate (SDS). Thiocholine, the reaction product, reacts with DTNB, forming 5-thio-2-nitrobenzoate, whose optical adsorption was then read at 412 nm. The enzyme concentration was kept constant and set to produce an optimal spectrophotometric signal. The physiological effects were quantified as the AChE inhibition, which is given as follows:

(1) $$I=100\times \frac{A_{0}-A}{A_{0}}$$

where A_0_ and A stand for the AChE activity in the absence of OP and the one measured after exposure to a given OP.

## 3. Results and Discussion

### 3.1. Physicochemical Characterization of SCGs

#### 3.1.1. Particle Sizes, Morphology, and Chemical Composition

**Figure 1:**
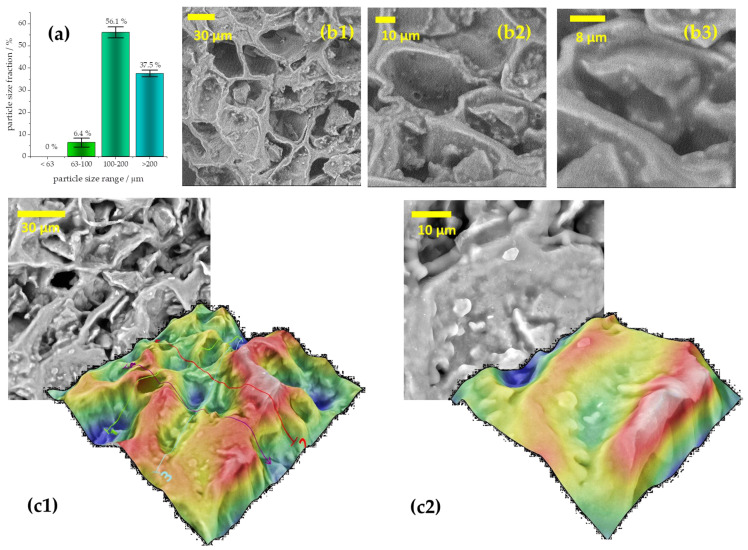
(**a**) Particle size distribution obtained by fractional sieving; (**b1**–**b3**) SEM micrographs of SCGs at magnifications ×2000, ×5000, and ×10,000 (from left to right); (**c1**,**c2**) 3D surface reconstruction of SCG powder under magnifications ×2000 and ×5000.

The particle size distribution, determined by the fractional sieving method (Figure 1a), shows that the largest fraction of SCG particles is in the 100–200 μm range. Particle sizes in the order of 10^2^ μm generally agree with previous reports of SCG properties, but the SCG particles reported here are somewhat smaller than in earlier reports [30,31], likely due to the grounding step we performed. We note that particle sizes of the same order of magnitude are frequently reported for biobased carbon materials used as adsorbents (see, for example, [32]). However, carbon materials typically have large specific surface areas (SSA) due to the developed internal pore structure. SSA is usually determined by gas adsorption measurements, but in our case, a reliable determination of SSA was not possible, and this is suspected to be due to very low SSA. Indeed, one of the scarce literature reports that claims the SSA of SCGs indicated the SSA in the range from 0.19 to 2.3 m^2^ g^−1^ [33]. 

To investigate the morphology of SCGs, SEM was used. In addition, the chemical composition of the SCGs was investigated using EDX. The results are presented in Figure 1. The SEM micrograph showed that SCGs have a sponge-like, porous, heterogeneous structure (Figure 1(b1–b3)). Similar morphologies and large pores with diameters of approx. 30 μm were reported previously [34,35]. It can be seen that the cavities are randomly distributed on the materials’ surface, with different size openings. SEM images were taken without deposition of the conductive layer on the SCGs sample, resulting in charge accumulation at higher magnifications. However, smooth pore walls can be observed without bright spots, indicating heavy elements in the sample. The surface 3D SEM reconstruction confirms these findings (Figure 1(c1,c2)). At a lower magnification (×2000), a rough surface profile is seen, with macropores entering the interior of the particles and contributing to the overall internal pore structure. However, higher magnification at the pore wall (×5000) indicates a rather smooth surface. 

**Figure 2:**
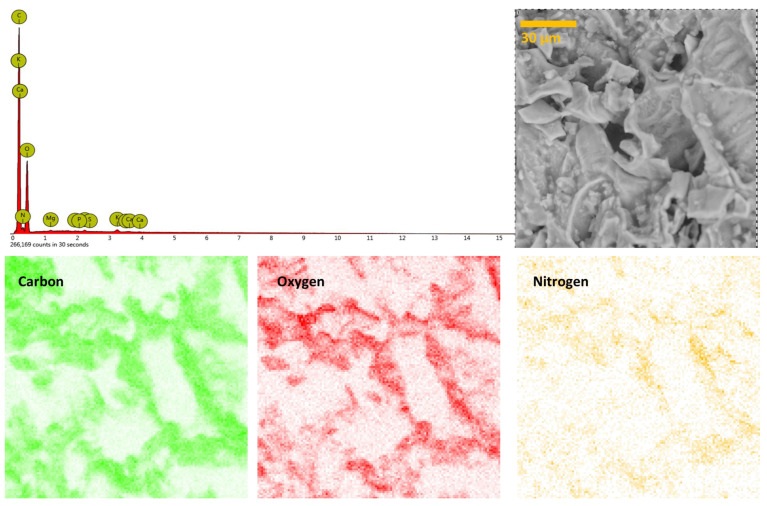
(**Top row**) EDX spectra of the indicated region (taken at a magnification of ×2000, 15 keV); (**bottom row**) EDX maps of carbon, oxygen, and nitrogen (64 × 64 pixels).

The EDX analysis showed that C, O, and N are present in high percentages in SCGs, namely 62.6 at. %, 28.1 at. %, and 9.0 at. %, respectively. Besides those mentioned, Mg, P, Ca, K, and S are present in traces (Figure 2, top row), constituting less than 0.3 at. % of the SCG sample. These elements are uniformly distributed in the sample, as indicated by the EDX maps (Figure 2, bottom row). The EDX analysis agrees with the literature and elemental composition of SCGs, as the other research reported a similar elemental composition with a different order of trace elements from source to source [36,37]. Here, we found that the trace elements’ composition (in at. %) follows the order K (0.09) > S = Mg (0.05) > P = Ca (0.02). We note that a previously low N content (<2%) was reported for SCGs [10], but using the elemental analysis, which also gives the H content, thus effectively reducing the contents of the other elements in the samples; meanwhile, here, H cannot be determined using EDX. 

#### 3.1.2. Surface Functional Groups of SCGs 

**Figure 3:**
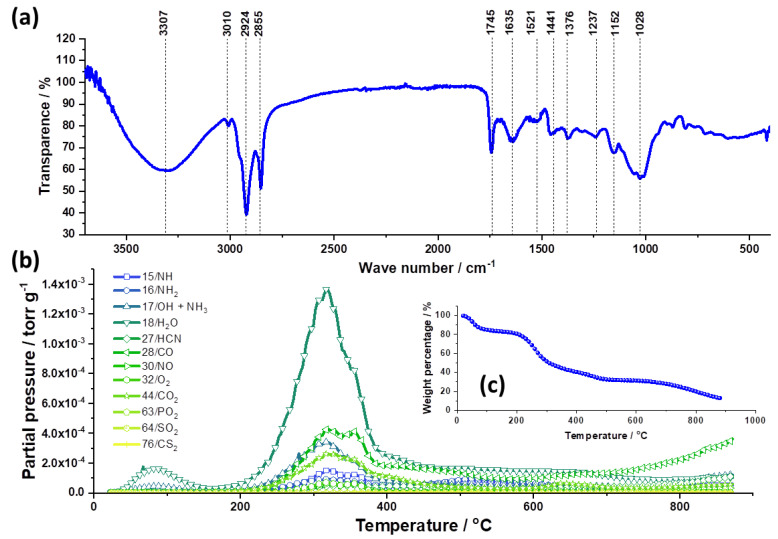
(**a**) ATR-FTIR spectrum of SCGs. (**b**) TPD curves of most common functional groups and molecules in SCGs. Inset (**c**) gives the TGA curve of SCGs under a helium atmosphere.

The FTIR spectrum of the SCGs is shown in Figure 3a. The obtained FTIR spectrum correlates well with the literature and assumed compounds in SCGs [36,37,38]. The bands present in the FTIR spectrum of the SCGs are assigned to a specific vibration. The broad band on 3307 cm^−1^ was attributed to the stretching of the O-H group due to hydrogen bonding. Bands at 3010 cm^−1^ and 2924 cm^−1^ indicated the presence of C-H bonds where C is sp^2^ and sp^3^ hybridized, respectively. The sharp band on 1745 cm^−1^ was assigned to C=O stretch vibration, while the 1635 cm^−1^ band was attributed to C=N stretch vibration. The presence of a condensed aromatic system is assumed due to bands on 1521 cm^−1^ and 1441 cm^−1^ representing aromatic skeletal stretch and aromatic vibrations coupled with aromatic C-H in-plane vibrations, respectively. It is a clear indication of ligning presence, as found in Ref. [10]. An aliphatic C-H stretch vibration was assigned to the band on 1376 cm^−1^. The deformation vibration of the C-N and C-O bands in secondary alcohols and C-N are assigned to bands on 1237 cm^−1^ and 1152 cm^−1^. The band on 1028 cm^−1^ was attributed to the coupling of aromatic C-H in-plane deformation vibrations and C-O stretch vibrations in primary alcohols.

The TGA curve (Figure 3c) shows that by increasing the temperature from 20 to 900 °C in the helium atmosphere, SCGs go through three significant weight losses. In the first stage, from 20 to 85 °C, SCGs lose about 15% of their weight. The second stage starts at 200 and ends at 500 °C. It is the stage that represents carbonization, and during this stage, SCGs lose 48% of their initial weight. The third stage, during which SCGs lose another 15% of their initial weight, starts at 670 °C and lasts up to 900 °C. At the end of the heating process, 12% of the initial SCGs’ weight is left.

The TPD curves for the most common functional groups and molecules desorbed during the heating process of SCGs are shown in Figure 3b. The same trend that TGA showed can be seen on these curves. In the correlation of TGA and TPD, it can be concluded that, during the first stage (20–85 °C), SCGs release physisorbed water. During the phase of SCG carbonization (200–500 °C), the weight loss is primarily due to the release of chemisorbed water, CO, and CO_2_ molecules. Besides these, it can be noticed that all of the investigated functional groups and molecules are desorbed in this stage. During the third stage (670–900 °C), additional CO and CO_2_ molecules are released, leading to the final weight loss. The evolved functional groups agree well with the elemental content found by EDX and also with the FTIR spectra of SCGs. A high concentration of H is expected in the sample, in line with Ref. [10], originating from physisorbed and strongly bound water, OH and NH_2_ groups, and terminal C-H bonds. 

### 3.2. Adsorption of Chlorpyrifos and Malathion on Spent Coffee Grounds

#### 3.2.1. Kinetics of Adsorption 

To investigate the kinetic parameters of the adsorption process, 1 mg mL^−1^ of SCG suspension was incubated with CHP and MLT in the concentration 5 × 10^−5^ mol dm^−3^ for various time intervals (from 1 min to 24 h) at 25 °C. The concentration of adsorbed pesticides was calculated as a difference between their initial concentration (C_0_) and the equilibrium concentration (C_e_) measured with UPLC after removing the adsorbent. The kinetics of CHP and MLT adsorption onto SCGs were analyzed using the nonlinear pseudo-first, pseudo-second, Elovich, and intraparticle diffusion models. The corresponding equations are listed in Table 1.

In Table 1, parameter q_t_ represents the amount of adsorbed OP at time t (mg g^−1^), while q_e_ is the amount of adsorbed OP at equilibrium (mg g^−1^). Parameter k_1_ stands for the adsorption rate of pseudo-first order (min^−1^), k_2_ is the adsorption rate of pseudo-second order (g mg^−1^ min^−1^), α is the initial adsorption rate in the Elovich model (mg g^−1^ min^−1^), and β is desorption constant in the Elovich model (g mg^−1^). Parameter k_id_ represents the adsorption rate constant of the intraparticle diffusion model (mg g^−1^ min^−0.5^), and C is connected to a boundary layer (mg g^−1^).

**Figure 4:**
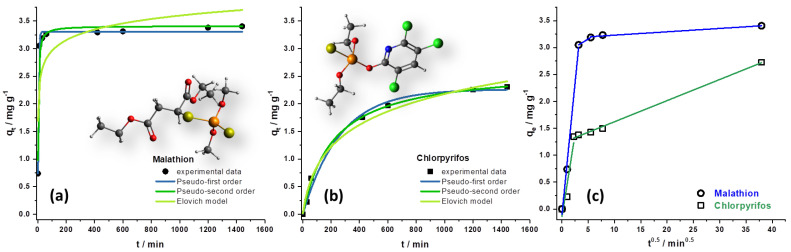
Graphical representation of kinetic models for the adsorption of MLT (**a**) and CHP (**b**) on SCGs. (**c**) Fitting experimental data into the intraparticle diffusion kinetic model for MLT and CHP. Insets present the optimized molecular structures of MLT and CHP (color legend for atoms: dark gray—C; red—O; yellow—S; orange—P; blue—N; green—Cl; and white—H).

The nonlinear forms of the mentioned kinetic models and the intraparticle diffusion model fittings are shown in Figure 4. The kinetic parameters and corresponding R^2^, χ^2^, and Root Mean Square Error (RMSE) values are given in Table 2.

By observing the results presented in Figure 4, it can be concluded that the adsorption equilibrium is reached after 10 min for MLT and 1440 min for CHP. In addition, it can be supposed that in the case of both MLT and CHP adsorption, the pseudo-first-order kinetic model fitted the experimental data better compared to the pseudo-second and Elovich models, according to the R^2^ and χ^2^ values. The calculated adsorption capacities of SCGs under experimental conditions are 3.31 mg g^−1^ for MLT and 2.26 mg g^−1^ for CHP. A higher k_1_ value for MLT adsorption indicates a higher adsorption rate than that of CHP. 

The value α, representing the initial adsorption rate, and the value β, the desorption constant, obtained from the Elovich model constant also indicate that the adsorption of CHP is noticeably slower than the adsorption of MLT on SCGs, as the α value is lower than the β value in the case of CHP adsorption.

By plotting q_t_ against t^0.5^, three linear stages for MLT and two linear stages for CHP could be clearly observed in the intraparticle kinetic model (Figure 3c). The breakpoints for MLT adsorption onto SCGs are 10 min and 60 min, and for CHP, 10 min. The first rapid stage is attributed to the diffusion of MLT and CHP molecules through the solution to the external surface of the SCGs [39]. The second stage represents intraparticle diffusion, in which MLT and CHP molecules diffuse into the pores of the SCGs. The third stage seen for MLT is attributed to the final equilibrium stage. The K_id_ values decrease by one order of magnitude after the breakpoints, indicating slower adsorption in every subsequent stage. The C value, representing the boundary layer, increases, suggesting that the boundary layer has a high significance in the adsorption of MLT and CHP onto SCGs. It can be concluded that the three mentioned processes control the rate of molecule adsorption for MLT and two for CHP, but for each time range during the adsorption process, one process at a time determines the adsorption kinetics. We believe that differences in the molecular structure of MLT and CHP, the first being aliphatic, while the other one possessing aromatic moiety (Figure 4, insets), renders drastically different adsorption kinetics. As CHP is a less polar molecule, also reflected in much lower solubility compared to MLT, this property likely reduces its adsorption rate on the solvated surface of SCGs. It also slows down its diffusion into pores, as stage two in the intraparticle diffusion model for CHP extends over 1000 min without reaching the final equilibration stage. 

#### 3.2.2. Adsorption Isotherms

To further study the adsorption process, 1 mg mL^−1^ of SCGs was incubated with MLT and CHP in the concentration range from 5 × 10^−6^ to 1 × 10^−4^ mol dm^−3^ for 24 h at 25, 30, and 35 °C. The obtained data were fitted with several nonlinear isotherm models (Freundlich, Langmuir, Temkin, and Dubinin–Radushkevich (DR)). The corresponding equations for used isotherms are given in Table 3.

In Table 3, the q_t_ parameter represents the amount of adsorbed OP for a given C_e_ (mg g^−1^), C_e_ is the equilibrium concentration (mg dm^−3^), and K_F_ is the Freundlich isotherm constant related to the adsorption capacity ((mg g^−1^)(dm^3^ g^−1^)^1/n^). Parameter n stands for the so-called adsorption intensity, q_max_ represents the maximum amount of adsorbed OP (mg g^−1^), and K_L_ is the Langmuir isotherm constant (dm^3^ mg^−1^). R is the universal gas constant (8314 J K^−1^ mol^−1^), T is the temperature (K), and b_T_ represents the Temkin isotherm constant (J g mol^−1^ mg^−1^), and K_T_ is the Temkin isotherm equilibrium binding constant (dm^3^ mg^−1^). At the same time, q_DR_ is the theoretical isotherm saturation capacity (mg g^−1^), while K_DR_ (mol^2^ J^−2^) is the Dubinin–Radushkevich isotherm constant. The Polanyi potential, ε (J mol^−1^), is calculated from C_e_, while K_DR_ is related to the adsorption mean free energy (E), as E = 1/(2K_DR_)^1/2^.

**Figure 5:**
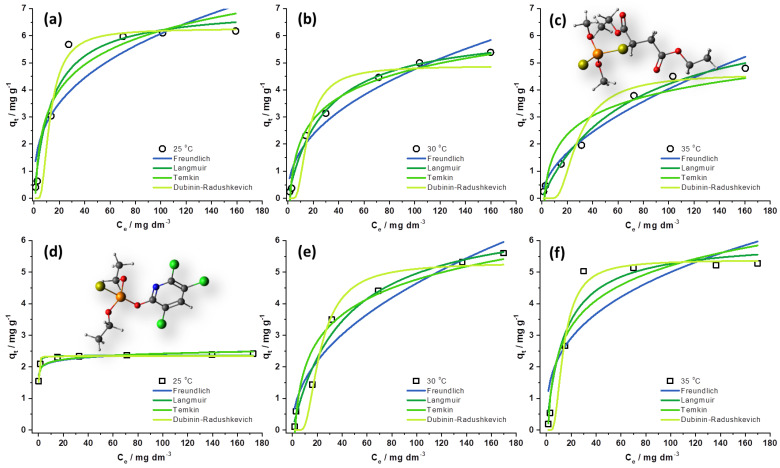
Top row: graphical representations of isotherm models for MLT adsorption on SCGs at (**a**) 25 °C, (**b**) 30 °C, and (**c**) 35 °C. Bottom row: graphical representations of isotherm models for CHP adsorption on SCGs at (**d**) 25 °C, (**e**) 30 °C, and (**f**) 35 °C.

Nonlinear forms fitting of all investigated isotherms are shown in Figure 5. The parameters obtained from the different models provide essential information on the adsorption mechanisms and the surface properties and affinities of SCGs towards MLT and CHP. Table 4 summarizes the parameters obtained from the nonlinear plots of each isotherm. The fitting of the models to the experimental data was evaluated by χ^2^ and R^2^. The R^2^ values are the highest (R^2^ > 0.9), while the χ^2^ and RMSE values are the lowest for the Langmuir model, suggesting that that model could best describe the experimental data. Still, most of the used isotherm models fit with most experimental data, so the calculated parameters are relevant for discussion.

The n-value of the Freundlich isotherm model for MLT adsorption is higher than 1, indicating that the adsorption is a favorable process. The decrease of the n-value with the increase in temperature indicates that the affinity of the adsorbent for MLT declines with the rising temperature. As mentioned, the Langmuir model showed the best agreement with the experimental results, indicating that the monolayer of MLT is adsorbed on an energetically homogenous surface without the interaction between adsorbed molecules. It indicates that all active sites are energetically equivalent and that equilibrium is reached when a monolayer is formed. Even though the temperature increase had almost no impact on the maximum amount (q_max_ = 7.16 mg g^−1^) of MLT that could be adsorbed by SCGs, with the decrease of the K_L_ value, it can be concluded that the interaction between MLT and SCGs was weakened. It is also confirmed by the decrease of the E value obtained from the Dubinin–Radushkevich isotherm model. As E << 8000 J mol^−1^, the proposed mechanism of MLT adsorption on SCGs is likely to be physisorption [40]. The Temkin isotherm model showed that the b_T_ values, which are related to adsorption heat, increase with the temperature. It could indicate that the molecules of MLT are more readily adsorbed onto the SCGs’ surface, contrasting with the previous parameters obtained from the rest of the tested isotherms. However, since the χ^2^ and RMSE values are higher for the Temkin isotherm than other investigated models, it is unsurprising that the findings provided different indications.

In the case of CHP adsorption, the n-values obtained from Freundlich isotherm models are also above 1, again indicating the favorability of the adsorption process. In addition, the n-value decreases with the temperature increase. The affinity of the adsorbent for CHP is declining with the rising temperature. Still, the n-values should be taken cautiously regarding the not-high R^2^ values for all temperatures (Table 4). Similar to the results for MLT adsorption, the Langmuir isotherm model provides the best fit for adsorption at all temperatures. In theory, that indicates that CHP is adsorbed on an energetically homogenous surface without the interaction between adsorbed molecules in the form of the monolayer. Adsorption of CHP onto SCGs at 25 °C provides the highest K_L_ value (K_L_ = 9.23 dm^3^ mg^−1^). It is in agreement with the n-values from the Freundlich isotherm. The obtained qmax values rise with the increasing temperature, and the maximum adsorption capacity is calculated to be 7.00 mg g^−1^. The energy of adsorption obtained from the DR isotherm model indicates that the adsorption mechanism is physisorption. Still, at the temperature of 25 °C, there is a strong indication that the chemisorption also occurs. The Temkin isotherm model showed that the b_T_ values decreased with the temperature increase, meaning that the readiness of the CHP molecule for adsorption on SCGs was decreasing, which agrees with the other calculated parameters.

#### 3.2.3. Thermodynamic Analysis

The thermodynamic parameters of adsorption provide information about the interactions between the adsorbate molecules and the surface. The most important thermodynamic adsorption parameters are the enthalpy (ΔH), entropy (ΔS), and Gibbs free energy (ΔG) of the adsorption process. These parameters give an indication of the heat, randomness changes, and spontaneity of the process. However, we note that the exact values of the thermodynamic parameters strongly depend on the choice of the standard state, as discussed in Ref. [41]. Thus, we adopted the recommendations for the standard state by Chen et al. [41].

The mentioned thermodynamic parameters are linked through the Gibbs–Helmholtz equation:

(2) $${\Delta G}^{0}={\Delta H}^{0}-{T\Delta S}^{0}$$

To determine the ΔH^0^ and ΔS^0^ values to calculate ΔG^0^, it is necessary to plot the Van’t Hoff equation, assuming that ${\Delta G}^{0}=-{\mathrm{RTlnK}}_{\mathrm{dist}}^{0}$:

(3) $${\mathrm{lnK}}_{\mathrm{dist}}^{0}=-\frac{{\Delta H}^{0}}{\mathrm{RT}}+\frac{{\Delta S}^{0}}{R}$$

where the standard distribution coefficient is defined by the following:

(4) $$K_{\mathrm{dist}}^{0}=\frac{q_{e}}{C_{e}}\times \frac{C^{0}}{q^{0}}$$

**Figure 6:**
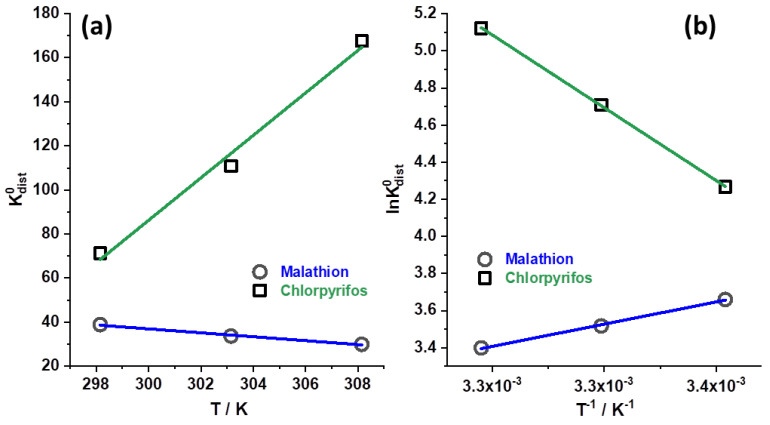
(**a**) Graphic representations of the amount of MLT (starting concentration 5 × 10^−4^ mol dm^−3^) and CHP (starting concentration 1 × 10^−4^ mol dm^−3^) dependence on temperature. (**b**) Van’t Hoff plot.

The values of C^0^ and q^0^ define the standard state for OP in solution (1 mol dm^−3^) and in the adsorbed state (1 mol kg^−1^), rendering the standard distribution coefficient to dimensionless quantity. Figure 6a shows graphic representations of the amount of MLT and CHP dependence on temperature and Van’t Hoff plot (Figure 6b). The extracted thermodynamic parameters for the adsorption of MLT and CHP onto SCGs are summarized in Table 5.

**Figure 7:**
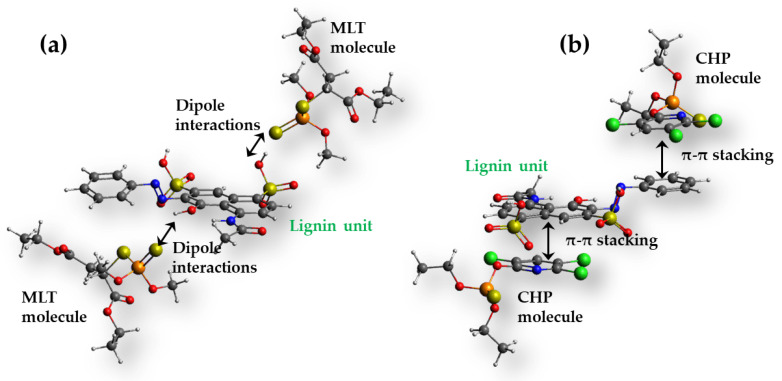
Schematic presentation of lignin monomer unit (structure data taken from [43] with (**a**) two MLT molecules via dipole interactions and (**b**) two CHP molecules via π-π stacking interactions. Structures were optimized by molecular mechanics in Avogadro [44] with an MMFF94 force field [45].

Figure 6a shows that increasing the temperature decreases the distribution coefficient of MLT, while the distribution coefficient of CHP increases. Temperature dependence is linear in both cases, but the effect of temperature is more pronounced for CHP. The obtained thermodynamic parameters (Table 5) explain this behavior and indicate that the adsorption of MLT is an exothermic process and that the adsorption is followed by a small negative change of entropy. Thus, for MLT, the adsorption on SCGs is an enthalpy-driven process, compensating for the reduction in entropy, giving ΔG^0^ negative, and increasing with temperature. On the other hand, the case of CHP adsorption onto SCGs is the opposite, as adsorption is an endothermic process, but the adsorption process is followed by a large positive entropy. Hence, CHP adsorption onto SCGs is an entropy-driven process. Again, the Gibbs free energy change is negative (slightly more negative than for MLT) and decreases with temperature, suggesting spontaneous adsorption, in line with experimental findings. Considering that a large fraction of the SCGs is constituted of lignin [10], we believe that the differences in thermodynamics of MLT and CHP adsorption can be explained by different types of interactions between SCGs and the studied OPs, keeping in mind that the adsorption process was classified as physisorption. We suggest that, in the case of MLT, dipole and dispersion interactions (Figure 7a) dominate the adsorption, thus becoming less prominent as the temperature increases. Such interactions can be placed between MLT molecules and different functional groups observed by FTIR (Figure 3a) and TPD. In the case of CHP, relatively strong π-π stacking between the aromatic ring of CHP and phenolic moieties of lignin can also take place (Figure 7b). However, in this case, desolvation of the surface has to take place, resulting in a positive change of the enthalpy and a large increase in entropy, resulting in negative Gibbs free energy change. As mentioned in Section 3.2.1., CHP and MLT show drastically different kinetic behavior, which was hypothesized to originate from different structures of these two pesticides. The argument mentioned above and the results regarding thermodynamic functions of the adsorption process support previous assumptions. In the case of CHP, it is proposed that desolvation of the SCG surface slows the adsorption down when compared to the MLT adsorption. As the dipole interactions are electrostatic, thus decaying with the distance but generally reaching longer ranges then π-π stacking (operative at distances below 4 Å [42]), it is assumed that MLT adsorption can proceed without significant slowdown due to desolvation. At the same time, the molecular forces capture MLT molecules at more considerable distances compared to CHP.

### 3.3. Application of SCGs in Food Processing—Plant Extracts Testing and Eco-Neurotoxicology Assessment

To assess the potential for SCGs’ application in food processing, the material was tested as an adsorbent in plant extracts. As mentioned, MLT is used to treat lemon crops, and CHP is used in mint crops. Therefore, diluted lemon juice for MLT and mint extract for CHP were prepared as described in Section 2.4. The samples were spiked with respective OP in the concentration of 5 × 10^−5^ mol dm^−3^. For comparison, the adsorptions of MLT and CHP from deionized water and 50% ethanol, respectively, were investigated under identical conditions. The results are shown in Table 6.

Our primary goal was to reduce the toxicity of plant extract samples and confirm that no respective oxo-analogs are formed during adsorption. To estimate the eco-neurotoxicity of the samples before and after the adsorption of OPs on SCGs, the AChE inhibition was measured, as described in Section 2.5. AChE inhibition is an important benchmark for eco-neurotoxicity, as it indicates a general impact of contaminants on the species containing AChE in their nervous systems [46]. The results are also summarized in Table 6.

From the data presented in Table 6, it is obvious that the matrix effects in plant extracts tested in this work have a minor impact on the OPs’ uptake. The amount of the adsorbed pesticides did not significantly change in the plant-extract analysis compared to deionized water and ethanol, indicating that SCGs as the adsorbent maintain a good performance and can be successfully used in plant extracts’ treatment. Moreover, the AChE inhibition due to the contact with samples was reduced after the adsorption in all cases, indicating that no more toxic products, such as OPs’ oxo-forms, were formed during this process. While the reduction of toxicity is not as prominent as in the case of some much more potent adsorbents, such as carbon materials [47,48], we note that SCGs’ performance falls well within the range of previously reported biowaste adsorbents and surpasses many of them in terms of MLT and CHP adsorption capacity, as is discussed further. Moreover, an important advantage is that such an adsorbent is truly green and sustainable, as it does not require a carbonization step during which large amounts of CO_2_ are released. In fact, with adsorption capacities in the order of 10^0^ mg g^−1^, SCGs perform quite similarly to carbons derived from biomass if the capacity is calculated versus raw material (precursor). Namely, during the carbonization and activation steps, a large amount of the precursor is lost, giving a yield in the order of 10^0^–10^1^% [49]. Thus, carbon materials with typical capacities for OPs’ removal around 100 mg g^−1^ under high and realistic adsorbent loadings [50] have adsorption capacities in the order of 10^0^ mg g^−1^ when referring to the raw precursor mass. Another critical point is the actual affinity of adsorbents towards contaminants. Here, the presented SCGs have a very low SSA (the direct measurement was not possible), but the adsorption capacities are in the order of 10^0^ mg g^−1^. Carbon-based adsorbents, on the other hand, have SSAs in the order of hundreds of m^2^ g^−1^. Thus, the affinity of the SCG surface towards MLT and CHP is quite similar to that of carbon materials, resulting from specific chemical composition and surface chemistry (Figure 7). Hence, as the adsorbent surface, not the adsorbent mass, is responsible for the adsorption process, the guidelines for improving SCGs’ (mass normalize) adsorption capacities are straightforward, increasing SSA while maintaining surface chemistry unchanged. In the next section, in order to put the obtained results in a proper context, we compare the performance of the here-described SCGs with previously reported biowaste-based adsorbents for MLT and CHP.

### 3.4. Biowaste as Adsorbent for Different Pollutants—Evaluation of SCGs’ Performance Compared to the Data Available in the Literature

SCGs have been studied for the adsorption of various water contaminants. The removal of Cd using SCGs was investigated by Patterer et al. [51] and Kim and Kim [52]. Patterer et al. concluded that the adsorption of Cd is an exothermic and spontaneous process and that the adsorption capacity is 4.484 mg g^−1^ [51]. In their research, Kim and Kim reported that the adsorption of Cd is irrelevant to the pH values of the solution in the range of 4–8, while the adsorption capacity of the SCGs was 19.32 mg g^−1^ [52]. Besides heavy metals, SCGs are shown to be excellent adsorbents of dyes. In their work, Block et al. found that non-treated SCGs are a promising adsorbent for methylene blue, removing 80% of the dye in 3 h of contact but adsorbing only 20% of methyl orange, making it a partly selective adsorbent [53]. 

Many sustainable materials can be effectively used as adsorbents for CHP. For example, Memon et al. successfully used walnut shell powder to remediate chlorpyrifos. The adsorption efficiency increased with the increase in temperature, similar to the here-reported behavior of SCGs. The used material had an adsorption capacity of 0.99 mmol g^−1^ (347 mg g^−1^) [54]. Moreover, Rojas et al. investigated the adsorption of chlorpyrifos on sunflower seed shells, rice husk, composted sewage sludge, and soil and concluded that their adsorption capacities are 0.035 mg g^−1^, 0.014 mg g^−1^, 0.010 mg g^−1^, and 0.02 mg g^−1^ respectively [55]; thus, two orders of magnitude below here reported capacities of SCGs towards CHP. 

Apart from CHP, research has been conducted on MLT remediation, but to a much lesser extent. The biosorption of MLT from aqueous solutions was investigated using two types of herbal leaf powder by Yadamari et al. The adsorption capacity for *Achyranthes aspera* was 3.401 mg g^−1^, and for *Phyllanthus niruri*, it was 2.664 mg g^−1^ [56]. Veličković et al. reported the adsorption capacity of malathion on pulverized river shellfish shells of 46.462 mg g^−1^. The adsorption of malathion in the mentioned investigation was assessed as exothermic, similar to the results presented in this work [57]. A comparative analysis of the maximum adsorption capacity obtained in this work and the previously mentioned research available in the literature is given in Table 7.

From the presented data, it can be seen that SCGs are a reasonably good adsorbent for MLT and CHP remediation. Moreover, the obtained adsorption capacities are comparable with the previously reported MLT and CHP adsorption capacities on different types of biowastes and raw materials. Considering the discussion provided in the previous section regarding the performance of carbon-based adsorbents, losses of precursor mass, and significant CO_2_ evolution during the carbonization process, we believe that SCGs have the potential for practical application in remediation processes, as they effectively stand side by side with apparently much more efficient adsorbents. However, we acknowledge that a detailed (socio-)techno-economical assessment is needed to correctly judge the possible use of SCGs in environmental remediation processes. 

## 4. Conclusions

In this work, MLT and CHP adsorption on SCGs from aqueous solutions was analyzed. The adsorption kinetics was best described by the pseudo-first-order kinetic model for both MLT and CHP adsorption, while MLT adsorption was found to be significantly faster compared to CHP. The Langmuir isotherm model best describes the adsorption process for both pesticides, giving the maximal adsorption capacity of 7.16 mg g^−1^ for MLT and 7.00 mg g^−1^ for CHP; while based on the Dubinin–Radushkevich isotherm model, the adsorption process can be safely classified as physisorption. MLT adsorption on SCGs was found to be exothermic, whereas CHP adsorption is endothermic and entropy driven. The adsorption efficiency of MLT and CHP using SCGs in a complicated matrix of fruit extracts remained constant. The eco-neurotoxicity results showed that no more toxic products were formed during adsorption, indicating that SCGs are a safe-to-use adsorbent for pesticide removal. Their benefits as an adsorbent should be considered in line with the fact that their adsorption performance is similar to carbon materials if referred to the precursor mass used for carbon production while completely removing complicated steps involving carbonization and activation, resulting in significant CO_2_ emissions. Considering that SCGs are globally available, their use in the remediation process also alleviates the problem of waste management, being particularly beneficial for less-developed countries, which, as a rule, suffer from extensive pesticide usage. 

## Figures and Tables

**Table 1:** Kinetic models’ equations.

Kinetic Model	Equation
Pseudo-first-order model	$q_{t}=q_{e}\left(1-e^{-k_{1}t}\right)$
Pseudo-second-order model	$q_{t}=\frac{q_{e}^{2}k_{2}t}{1+q_{e}k_{2}t}$
Elovich model	$q_{t}=\frac{1}{\beta }\ln\left(1+\alpha \beta t\right)$
Intraparticle diffusion model	$q_{t}=k_{\mathrm{id}}t^{0.5}+C$

**Table 2:** Kinetic parameters for MLT and CHP adsorption on SCGs.

Parameter	MLT	CHP
**Pseudo-first order**		
q_e_ (mg g^−1^)	3.31 ± 0.03	2.26 ± 0.07
k_1_ (min^−1^)	0.25 ± 0.02	0.0038 ± 0.0005
χ^2^	0.004	0.006
R^2^	0.997	0.994
RMSE	0.0714	0.0964
**Pseudo-second order**		
q_e_ (mg g^−1^)	3.41 ± 0.08	2.66 ± 0.08
k_2_ (mg min^−1^ g^−1^)	0.12 ± 0.03	0.0018 ± 0.0003
χ^2^	0.037	0.008
R^2^	0.970	0.993
RMSE	0.1720	0.0565
**Elovich model**		
α (mg g^−1^ min^−1^)	2.97 ± 0.23	0.018 ± 0.005
β (g mg^−1^)	1.47 ± 0.17	1.55 ± 0.18
χ^2^	0.215	0.011
R^2^	0.859	0.990
RMSE	0.5948	0.0579
**Intraparticle diffusion model**		
**part I**		
C (mg g^−1^)	0.00 ± 0.16	0.00 ± 0.28
K_id_ (mg g^−1^ min^−0.5^)	0.98 ± 0.08	0.61 ± 0.19
R^2^	0.987	0.921
**part II**		
C (mg g^−1^)	2.86 ± 0.01	1.23 ± 0.02
K_id_ (mg g^−1^ min^−0.5^)	0.060 ± 0.001	0.039 ± 0.002
R^2^	^−^	0.997
**part III**		
C (mg g^−1^)	3.17 ± 0.02	^−^
K_id_ (mg g^−1^ min^−0.5^)	0.0062 ± 0.0009	^−^
R^2^	0.963	^−^

**Table 3:** Adsorption isotherms’ equations.

Model	Nonlinear Form
Freundlich isotherm	$q_{t}=K_{F}C_{e}^{\frac{1}{n}}$
Langmuir isotherm	$q_{t}=\frac{q_{\max}K_{L}C_{e}}{1+K_{L}C_{e}}$
Temkin isotherm	$q_{t}=\frac{\mathrm{RT}}{b_{T}}{\mathrm{lnK}}_{T}C_{e}$
Dubinin–Radushkevich isotherm	$q_{t}=q_{\mathrm{DR}}e^{-K_{\mathrm{DR}}\varepsilon^{2}}$

**Table 4:** Adsorption parameters for MLT and CHP adsorption on SCGs.

	MLT			CHP		
T (°C) →	25	30	35	25	30	35
**Freundlich isotherm**						
K_f_ ((dm^3^ mg^−1^)^1/n^)	1.27 ± 0.54	0.64 ± 0.19	0.33 ± 0.10	1.90 ± 0.09	0.54 ± 0.20	1.07 ± 0.51
n	2.94 ± 0.84	2.29 ± 0.34	1.83 ± 0.22	19.2 ± 4.1	2.13 ± 0.37	2.98 ± 0.95
χ^2^	1.312	0.242	0.119	0.018	0.341	1.203
R^2^	0.808	0.946	0.967	0.808	0.934	0.771
RMSE	1.1456	0.4919	0.3456	0.1348	0.5840	1.097
**Langmuir isotherm**						
K_L_ (dm^3^ mg^−1^)	0.072 ± 0.023	0.035 ± 0.004	0.014 ± 0.002	9.23 ± 0.98	0.024 ± 0.006	0.07 ± 0.03
q_max_ (mg g^−1^)	7.06 ± 0.53	6.33 ± 0.21	7.16 ± 0.64	2.36 ± 0.03	7.00 ± 0.55	6.04 ± 0.52
χ^2^	0.316	0.022	0.045	0.003	0.108	0.323
R^2^	0.954	0.995	0.988	0.968	0.979	0.938
RMSE	0.5622	0.1485	0.2115	0.0550	0.3282	0.5687
**Temkin isotherm**						
K_T_ (dm^3^ mg^−1^)	0.92 ± 0.37	0.61 ± 0.10	0.52 ± 0.23	365000 ± 1200	0.50 ± 0.15	0.76 ± 0.33
b_T_ (J g mol^−1^ mg^−1^)	1800 ± 210	2170 ± 120	2600 ± 370	16700 ± 240	2070 ± 210	2130 ± 280
χ^2^	0.532	0.080	0.420	0.015	0.288	0.487
R^2^	0.922	0.982	0.885	0.845	0.944	0.907
RMSE	0.7293	0.2832	0.6479	0.1212	0.5367	0.6982
**Dubinin–Radushkevich isotherm**						
q_DR_ (mg g^−1^)	6.26 ± 0.22	4.89 ± 0.30	4.60 ± 0.38	2.34 ± 0.04	5.31 ± 0.25	5.39 ± 0.20
K_DR_ (mol^2^ J^−2^) × 10^5^	2.13 ± 0.41	3.15 ± 0.92	9.7 ± 3.4	0.0021 ± 0.0002	6.20 ± 1.20	2.31 ± 0.46
E (J mol^−1^)	153 ± 15	126 ± 19	72 ± 13	4850 ± 230	89.8 ± 8.7	147 ± 15
χ^2^	0.155	0.273	0.313	0.007	0.158	0.120
R^2^	0.977	0.939	0.914	0.928	0.969	0.977
RMSE	0.3937	0.5221	0.5597	0.0825	0.3978	0.3460

**Table 5:** Thermodynamic parameters of MLT and CHP adsorption for a SCG loading of 1 mg mL^−1^.

	MLT			CHP		
**T (°C)**	25	30	35	25	30	35
**ΔH^0^ (kJ mol** **^−^** **^1^** **)**	−(19.9 ± 0.8)			65.3 ± 0.7		
**ΔS^0^ (J mol** **^−^** **^1^** **K** **^−^** **^1^** **)**	−(36.5 ± 2.8)			254.6 ± 2.2		
**ΔG^0^ (kJ mol** **^−^** **^1^** **)**	−(9.1 ± 1.7)	−(8.8 ± 1.7)	−(8.7 ± 1.7)	−(10.6 ± 1.3)	−(11.9 ± 1.3)	−(13.1 ± 1.3)
**R^2^**	0.996			0.9997		

**Table 6:** OP concentrations and AChE inhibition in the tested samples (MLT in deionized water and lemon juice and CHP in 50% ethanol and mint ethanol extract) before and after the adsorption. Results were obtained when 1 mg mL^−1^ of adsorbent (SCG) was used at 25 °C.

Sample	[OP] (mol dm^−3^)		AChE Inhibition (% of Control)	
	Before the Adsorption	After the Adsorption	Before the Adsorption	After the Adsorption
MLT in deionized water	5.00 × 10^−5^	3.48 × 10^−5^	66 ± 5	45 ± 5
MLT in lemon juice	5.00 × 10^−5^	3.50 × 10^−5^	64 ± 5	50 ± 6
CHP in 50% ethanol	5.00 × 10^−5^	4.00 × 10^−5^	90 ± 4	81 ± 6
CHP in mint ethanol extract	5.00 × 10^−5^	4.15 × 10^−5^	92 ± 5	85 ± 5

**Table 7:** Maximum adsorption capacities for MLT and CHP adsorption for various biowastes and raw materials.

Material	OP	q_max_ (mg g^−1^)	Refs.
Walnut shell powder	CHP	347	[54]
Sunflower seed shells	CHP	0.035	[55]
Rice husk	CHP	0.014	
Composted sewage sludge	CHP	0.010	
Soil	CHP	0.020	
*Achyranthes aspera* leaves powder	MLT	3.401	[56]
*Phyllanthus niruri* leaves powder	MLT	2.664	
Pulverized river shellfish shell powder	MLT	46.462	[57]
Spent coffee grounds	MLT	7.157	This work
Spent coffee grounds	CHP	7.004	

## Data Availability

The data presented in this study are available on request from the corresponding author.
